# Economic burden of cholera in the WHO African region

**DOI:** 10.1186/1472-698X-9-8

**Published:** 2009-04-30

**Authors:** Joses M Kirigia, Luis G Sambo, Allarangar Yokouide, Edoh Soumbey-Alley, Lenity K Muthuri, Doris G Kirigia

**Affiliations:** 1 Health Financing and Social Protection Programme, World Health Organization, Regional Office for Africa, Brazzaville, Congo; 2 Regional Director, World Health Organization, Regional Office for Africa, Brazzaville, Congo; 3 WHO Representative, WHO Country Office, Kinshasa, Congo; 4 Information, Evidence and Research Programme, World Health Organization, Regional Office for Africa, Brazzaville, Congo; 5 Financial Advisor, Old Mutual Life Assurance Company Limited, Nairobi, Kenya; 6 University of New South Wales, School of Public Health and Community Medicine, Faculty of Medicine, Indigenous Health Unit, Sydney, Australia

## Abstract

**Background:**

In 2007, various countries around the world notified 178677 cases of cholera and 4033 cholera deaths to the World Health Organization (WHO). About 62% of those cases and 56.7% of deaths were reported from the WHO African Region alone. To date, no study has been undertaken in the Region to estimate the economic burden of cholera for use in advocacy for its prevention and control. The objective of this study was to estimate the direct and indirect cost of cholera in the WHO African Region.

**Methods:**

Drawing information from various secondary sources, this study used standard cost-of-illness methods to estimate: (a) the direct costs, i.e. those borne by the health-care system and the family in directly addressing cholera; and (b) the indirect costs, i.e. loss of productivity caused by cholera, which is borne by the individual, the family or the employer. The study was based on the number of cholera cases and deaths notified to the World Health Organization by countries of the WHO African Region.

**Results:**

The 125018 cases of cholera notified to WHO by countries of the African Region in 2005 resulted in a real total economic loss of US$39 million, US$ 53.2 million and US$64.2 million, assuming a regional life expectancies of 40, 53 and 73 years respectively. The 203,564 cases of cholera notified in 2006 led to a total economic loss US$91.9 million, US$128.1 million and US$156 million, assuming life expectancies of 40, 53 and 73 years respectively. The 110,837 cases of cholera notified in 2007 resulted in an economic loss of US$43.3 million, US$60 million and US$72.7 million, assuming life expectancies of 40, 53 and 73 years respectively.

**Conclusion:**

There is an urgent need for further research to determine the national-level economic burden of cholera, disaggregated by different productive and social sectors and occupations of patients and relatives, and national-level costs and effectiveness of alternative ways of scaling up population coverage of potable water and clean sanitation facilities.

## Background

In 2002, the world lost a total of 1 490 126 000 disability-adjusted life years (DALYs) from various diseases and conditions [1]. About 61 966 000 of the lost DALYs resulted from diarrhoeal diseases. An estimated 37.5% of the diarrhoea-related DALYs was lost in the World Health Organization (WHO) African Region alone.

Cholera is one of the main causes of diarrhoea. In 1997, a total of 118 349 cholera cases and 5 853 deaths were reported to WHO by countries of the African Region [2]. By the end of 2005, the number of cholera cases notified from the Region had increased to 125 018 (94.8% of the total 131 943 cholera cases reported globally). However, the number of cholera-related deaths reported from the Region had decreased to 2230 (98.2% of the 2272 cholera deaths reported globally) [3]. According to WHO [3], "Globally, the actual number of cholera cases is known to be much higher; the discrepancy is the result of underreporting and other limitations of surveillance systems, such as inconsistency in case definition and lack of a standard vocabulary (p.297)". The underreporting could be due to fear, among the notifying countries, of the potential negative impact on their tourism industry and export of commodities.

The etiological agent that causes cholera is *Vibrio cholerae*. The bacterial agent is associated with conditions that force populations to live under conditions of overcrowding, inadequate housing, inadequate excreta disposal systems, lack of potable water, floods, unhygienic human behavioural practices, poverty, civil unrest leading to internal displacement of people, and unhygienic food production, distribution and handling systems [4,5]. In sub-Saharan Africa, 63% of the population has no sustainable access to improved sanitation and 44% has no sustainable access to improved water sources [6]. The ever-increasing proportion of vulnerable African populations who live in the above-mentioned unsanitary conditions is constantly at risk of cholera outbreaks [2].

Prevention of cholera outbreaks entails mitigating the above-mentioned factors, especially assuring sustainable access to improved sanitation and water sources and observance of hygienic human behavioural practices. Cholera treatment entails rehydration with replacement of electrolytes lost. According to WHO guidelines [7], patients with: no dehydration should receive oral rehydration salts (ORS) at home; mild dehydration (who are restless and irritable, have sunken eyes, dry mouth, thirsty – drinks eagerly, skin pinch goes back slowly) should receive ORS and very close surveillance; severe dehydration (manifesting various symptoms – lethargic, unconscious, floppy, very sunken eyes, unable to drink, mouth very dry, skin pinch goes back very slowly, lack of tears among children) should receive intravenous therapy, antibiotics and ORS.

To the best of our knowledge, prior to the study reported in this paper, no other study has attempted to estimate the economic burden of cholera in the WHO African Region. This paper attempts to answer the question: from the patient's (family) and ministries of health perspective, what is the total cost of cholera in the African Region. The specific objectives were to estimate: (a) the direct costs, i.e. those borne by the health-care services and the families in directly addressing the cholera problem; and (b) the indirect costs, i.e. mainly the losses in productivity caused by the disease, borne by the individual, the family or the employer.

## Methods

### Data

The data on the number of cases notified to WHO in 2005, 2006 and 2007 were obtained from a WHO Database [8]. The unit costs of the "hotel" component (personnel, capital, utilities, maintenance, etc.) of hospitals and health centres were obtained from a WHO website [9]. Adam, Evans and Murray [10] provided the details of how the country-specific hospital and health centre "hotel" cost component was estimated. The standard treatment for mild/moderate and severe cholera was obtained from WHO guidelines [11]. Prices for medicines were obtained from the WHO/AFRO essential medicines price indicator document [12]. The average cost of a diagnostic test for cholera was collected purposively from private laboratory services in 10 Member States. The costs borne by households were obtained from the World Health Survey [13] and adjusted for inflation using information from the International Monetary Fund (IMF) Database [14]. The information on gross national income per capita was obtained from the World Bank database [15]. All the costs estimates for 2005, 2006 and 2007 were eventually expressed in 2002 prices using the average consumer price indices for the three years [14].

### Conceptual framework

**Figure 1:**
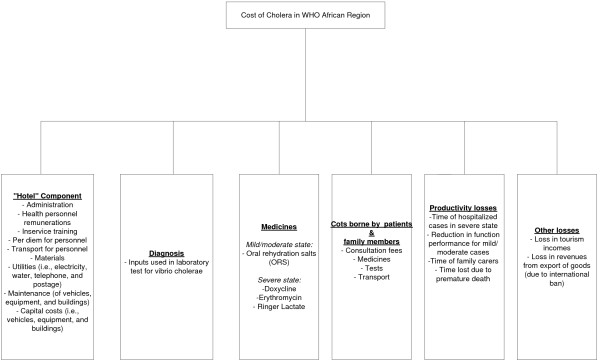
**Cost of cholera in WHO African region**.

Figure 1 presents an analytical framework of the cost of cholera in the WHO African Region. The framework consists of six components. The first component consists of the "hotel" hospital and health centre costs. It excludes cholera drugs and diagnostic tests. It includes the cost of administration, health personnel remunerations, in-service training, per diem and transport for personnel, materials, utilities (i.e. electricity, water, telephone, and postage), maintenance (of vehicles, equipment and buildings), and capital costs (i.e. vehicles, equipment and buildings) [9].

The second component consists of the cost of laboratory diagnosis of cholera cases. We assumed that the prices charged by private hospital laboratories for cholera diagnosis approximate the opportunity cost of resources used to perform laboratory test. This data was purposively collected by WHO Country Office health systems advisors in 10 countries, i.e. Burkina Faso, Congo, Ghana, Kenya, Malawi, Mauritania, Mauritius, Namibia, Senegal and Zambia.

The third component consists of the cost of medicines used to treat cholera cases. The estimations for medicines are based on the 125018 cases notified to WHO in 2005; 203564 cases notified in 2006; and 110837 cases notified in 2007 [8]. The standard treatment regimen for mild/moderate dehydration patients are ORS and very close surveillance [10]. According to a personal communication with the WHO Regional Adviser for cholera control programme, mild cholera cases will require on average about three litres of ORS solution over a period of about three days. The standard treatment regimens for severely dehydrated patients are summarized in Table 1.

**Table 1:** Standard treatment regimens for severe cholera cases

Antibiotics	Dose	Children			Adults (=>15 years)	Pregnant women
				
		Under 1 year	1–4 years	5–14 years		
Erythromycin 250 mg	30 mg/kg divided 4 times per day for 3 days	1/4 tablet 4 times/day 3 days	1/2 tablet 4 times/day 3 days	1 tablet 4 times/day 3 days	2 tablets 4 times/day 3 days	2 tablets 4 times/day 3 days
Doxycline	300 mg Single dose				3 tablets	
Ringer Lactate solution (IV) in litres	1 litre per day for 3 days	1 litre per day for 3 days	1 litre per day for 3 days	1 litre per day for 3 days	1 litre per day for 3 days	1 litre per day for 3 days

Source: WHO [11].

The fourth component consists of costs borne by patients and family members. The average cost borne by patient and family members was obtained from the World Health Survey [13] and adjusted for inflation rates in 2005, 2006 and 2007 [14] to obtain the costs for those years.

The fifth component is made up of productivity losses. These include: severe case's hospitalization time; reduction in function performance for mild/moderate cases; the time family carers spend accompanying cases to a health facility and visiting those that are hospitalized; and productive time lost due to premature death. Culturally, the majority of the people in African countries never retire. In the absence of social security systems covering entire populations, survival of the self-employed people largely depends on their ability to undertake their activities of daily living until they die. Even the few who are employed in the formal sector, after attaining official retirement age, they continue to lead economically active lives (in trade, farming, animal husbandry, etc.) for their remaining life expectancies. We did cost estimations for three scenarios, assuming people would work until they attain the age of (i) 73 years, i.e. the maximum life expectancy in the African Region (in Mauritius); (ii) 53 years, i.e. the average life expectancy in the Region; and (iii) 40 years, i.e. the minimum life expectancy in the Region (in Sierra Leone) [16]. Even for children (0–14 years) who die prematurely, we valued the years lost above 15 at the current gross national income per year, since they constitute a permanent loss from the future labour force. The productive years of life lost were discounted at a rate of 3% [17].

The last component consists of other losses to the affected African country's economies. Cholera outbreaks impact negatively on both domestic and international demand for tourism industry services of affected countries. There is evidence that when there are cholera outbreaks in African countries, developed countries usually discourage their citizens from traveling to those countries, which may reduce the numbers of tourists to affected countries. Consequently, that could lead to losses of revenues for the tourism industry, unemployment of people whose livelihood is dependent directly or indirectly on tourism, and reduction in tax revenues for the governments. In addition, it is not unusual to have international ban slapped on export of commodities from countries experiencing cholera outbreaks. When the latter happens, it may adversely affect the foreign exchange flows into the affected countries, which is likely to have many other externality costs.

The current study focuses on cost of "hotel" component, diagnosis, medicines, cost borne by patients and accompanying family members, and productivity losses. It was not possible to obtain the data needed to estimate the losses in tourism incomes and export revenues.

#### Cost Estimation

The total cost (TC) incurred by ministries of health, cholera patients and family members equals direct cost plus indirect cost (which is value of productivity time lost) plus intangible cost (including physical and psychological pain) [18,19].

#### Direct cost

The total direct cost (DC) is the sum of total costs borne by government in operating and organizing health facility services and the out-of-pocket expenses borne by patients, family members and relatives. The cost of operating and organizing health facility services is the sum of the costs of medicines; cost of personnel time, utilities, non-pharmaceutical supplies, materials, maintenance of capital inputs, acquisition of capital inputs (vehicles, equipment and buildings); and cholera tests.

### Cost of medicines

Medicines cost is the sum of the costs of medicines for treating all children with mild/moderate cholera at health centre; adults (>= 15 years) with mild/moderate cholera at health centre; all under-1-year-old children with severe cholera at a hospital; all children aged between 1 and 4 years with severe cholera at a hospital; all children aged between 5 and 14 years with severe cholera at a hospital; and all adults (i.e. 15 years and above) with severe cholera at a hospital.

The cost of medicines for treating children and adults with mild/moderate cholera at health centre (CMCM) is the product of daily dosage of ORS; number of days that ORS is administered; unit cost of ORS; and the number of children and adults with mild/moderate cholera.

The cost of medicines for treating children aged under-1-year-old, 1–4 years and 5–14 years, and adults (i.e. 15 years and above) with severe cholera at a hospital is the product of dosage of erythromycin (and Ringer Lactate); number of times a dosage of erythromycin is taken per day; number of times a dosage of erythromycin is taken per day; price per unit of erythromycin; and the number of patients in relevant age bracket with severe cholera. Simply, cost of medicines equals quantity consumed times the unit price.

### "Hotel" cost of component of health centre consultations and hospitalizations

The hospital cost per hospital stay and per outpatient visit represent the "hotel" component (HC), i.e. excluding drugs and diagnostic tests and including other costs such as personnel, capital and food costs. The "hotel" component of hospital costs were obtained from a WHO website [9].

Hotel cost equals total cost of mild/moderate cholera cases' outpatient department (OPD) consultations per year plus total cost of severe cholera patients' hospitalizations per year. The first component is obtained by multiplying total number of mild/moderate cases; number of OPD visits per mild/moderate patient per episode; and cost per mild/moderate OPD consultation. The second component is the product of total number of severe cholera patients hospitalized; average length of stay; and cost per inpatient/day.

### Cost of cholera diagnosis

Cost of cholera test equals total number of cholera cases times cost of one cholera test. Thus, we assumed that all the patients presenting at health centres and hospitals manifesting cholera symptoms would undergo laboratory diagnostic test to ascertain the cause.

### Costs borne by households

The World Health Survey provided the general average health care costs (consultation, medicines, tests and transport) borne by households in 16 African countries, i.e. Burkina Faso, Cote D'Ivoire, Congo, Comoros, Ethiopia, Ghana, Kenya, Malawi, Mauritania, Mauritius, Namibia, Senegal, Swaziland, South Africa, Zambia and Zimbabwe [13]. The average health care cost per person was multiplied by total number of cases notified in each year to obtain the total health care cost borne by households.

#### Indirect cost

The total indirect costs (IC) is the sum of total cost of productive time lost due to mild/moderate cholera; total cost of productive time lost due to severe cholera; total cost of productive time lost due to cholera-related premature mortality; and productivity loss due to the work time lost by relatives accompanying and visiting patients.

### Cost of premature cholera-related mortality

A total of 2230, 5281 and 2287 people died from cholera in 2005, 2006 and 2007 respectively [8]. The distribution of those deaths across the five age brackets was obtained by multiplying the total number of cholera deaths by the cholera-related probabilities of death (PD) from Murray and Lopez [20]. Those authors estimated that the average duration of each episode would last 0.02 years for all age brackets. Thus, the productive life years lost (PLYL) for each age bracket were obtained subtracting the sum of the average age of onset and average duration of the episode from the minimum, average and maximum life expectancies in the African Region, respectively.

Total cost of premature cholera-related mortality is sum of the cost of premature cholera-related mortality among persons aged 4 years and less, aged 5–14 years, aged 15–44 years, aged 45–59 years, and aged 60 years and above. The cost of premature cholera-related mortality among persons of specific age group is the product of number of deaths, total number of productive discounted life years lost (i.e. years above 14 years of age) and gross national income per capita per year (US$). The productive life years lost were discounted at a rate of 3% [17].

### Cost of productive time lost due to severe cholera

Total cost of productive time lost due to temporary disability from severe cholera episodes (CPTSC) is the sum of cost of productive time lost due to severe cholera among persons aged 5–14 years, 15–44 years, 45–59 years, and 60 years and over. The non-fatal illness time lost among patients aged 4 years and less was not costed.

The cost of productive time lost due to severe cholera among persons of various age groups was obtained by multiplying the total number of severe cholera cases, average duration lived with cholera and gross national income per capita per year.

### Cost of productive time lost due to mild/moderate cholera

Total productive time lost due to mild/moderate cholera episodes (CPTMC) is the sum of cost of productive time lost due to mild/moderate cholera among persons aged 5–14 years, 15–44 years, 45–59 years, and 60 years and over. The non-fatal illness time lost among patients aged 4 years and less was not costed.

The cost of productive time lost due to mild/moderate cholera episodes among persons of various age groups was obtained by multiplying the total number of mild/moderate cholera cases, average number of days lived with mild/moderate cholera and daily gross national income per capita.

### Cost of productive time lost by care-givers accompanying patients for treatment

The cost of the work time lost by accompanying/visiting relatives is a product of the number of cholera cases, number of persons travelling to a health facility (i.e. the accompanying relative), number of days spent visiting a health facility per person per year and daily gross national income per capita per day.

The assumptions used in estimating direct and indirect costs can be found in the Appendix [see Additional file 1]. All the costs for 2005, 2006 and 2007 were expressed in 2002 prices using appropriate consumer price indices from the International Monetary Fund database [14].

## Results

### Total costs

Table 2 presents three scenario estimates of the direct and indirect costs of cholera in the WHO African Region in 2005. This study estimated the real total economic loss attributable to cholera to have been US$38,958,750 assuming a minimum regional life expectancy of 40 years; US$53,240,859 assuming a regional average life expectancy of 53 years; and US$64,208,880 assuming a maximum regional life expectancy of 73 years. The latter estimate consisted of a total direct cost of US$5,537,200 (8.51%). Out of that direct cost, 3.35% consisted of cost of medicines used in treating children and adults suffering mild/moderate cholera state at health centre; 1.29% consisted of cost of medicines used in treating children and adults suffering severe cholera state at hospitals; 49.49% consisted of the "hotel" cost component of mild/moderate cholera cases consultations at health centres; 11.77% consisted of the "hotel" cost component of severe cholera cases hospitalization; 29.17% for cholera-related diagnostic test; and 4.93% for health care costs incurred by households (patients and their family members) in search of cholera treatment.

**Table 2:** Direct and indirect real cost of cholera (US$) in 2005 (at 2002 prices)

	*Scenario 1: At maximum life expectancy of 73 years*		*Scenario 2: At average life expectancy of 53 years*		*Scenario 3: At minimum life expectancy of 40 years*	
	
*Summary of direct costs of cholera*	*US$*	% of grand TC	*US$*	% of grand TC	*US$*	% of grand TC
***(A). Direct costs***						
(1) Annual cost of medicines used in treating children and adults suffering mild/moderate cholera state at health centre	185,334	0.29	185,334	0.35	185,334	0.48
(2) Annual cost of medicines used in treating children and adults suffering severe cholera state at hospitals	71,561	0.11	71,561	0.13	71,561	0.18
(3) "Hotel" cost component of mild/moderate cholera cases consultations at health centres	2,740,314	4.27	2,740,314	5.15	2,740,314	7.03
(4) "Hotel" cost component of severe cholera cases hospitalization	651,741	1.02	651,741	1.22	651,741	1.67
(5) Cost cholera-related tests all cholera cases	1,615,047	2.52	1,615,047	3.03	1,615,047	4.15
(6) Health care cost borne by households	273,201	0.43	273,201	0.51	273,201	0.70
***Total Direct Costs***	***5,537,198***	***8.62***	***5,537,198***	***10.40***	***5,537,198***	***14.21***
***(B) Indirect costs***						
(7) Cost of disablement among severe cases	63,879	0.10	63,879	0.12	63,879	0.16
(8) Cost of disablement among mild/moderate cases	531,242	0.83	531,242	1.00	531,242	1.36
(9) Cost of premature deaths	56,691,858	88.29	45,723,836	85.88	31,441,728	80.71
(10) Productivity loss for care-givers	1,384,702	2.16	1,384,702	2.60	1,384,702	3.55
***Total Indirect Costs***	***58,671,681***	***91.38***	***47,703,659***	***89.60***	***33,421,551***	***85.79***
**Grand total cost**	**64,208,880**	**100**	**53,240,859**	**100**	**38,958,750**	**100**

The indirect costs amounted to US$58,671,681 (91.39% of total loss) worth of productive time that was lost by people of the African Region due to cholera disease. Out of the total indirect cost, about 96.63% was attributed to premature cholera-related mortality; 0.11% to productive time lost among severe cases; 0.91% to productive time lost among mild/moderate cholera cases; and 2.36% to productive time lost by family care givers.

Table 3 portrays three scenario estimates of the direct and indirect costs of cholera in the WHO African Region in 2006. This study estimated the real total economic loss attributable to cholera to have been US$ 91,863,606 assuming a minimum regional life expectancy of 40 years; US$ 128,136,952 assuming a regional average life expectancy of 53 years; and US$ 155,993,261 assuming a maximum regional life expectancy of 73 years. The latter estimate was made up of US$8.6 million (5.5%) of direct cost and US$147.4 million (94.5%) of indirect cost.

**Table 3:** Direct and indirect real cost of cholera (US$) in 2006 (at 2002 prices)

	*Scenario 1: At maximum life expectancy of 73 years*		*Scenario 2: At average life expectancy of 53 years*		*Scenario 3: At minimum life expectancy of 40 years*	
	
*Summary of direct costs of cholera*	*US$*	% of grand TC	*US$*	% of grand TC	*US$*	% of grand TC
***(A). Direct costs***						
(1) Annual cost of medicines used in treating children and adults suffering mild/moderate cholera state at health centre	277,058	0.18	277,058	0.22	277,058	0.30
(2) Annual cost of medicines used in treating children and adults suffering severe cholera state at hospitals	107,950	0.07	107,950	0.08	107,950	0.12
(3) "Hotel" cost component of mild/moderate cholera cases consultations at health centres	4,096,527	2.63	4,096,527	3.20	4,096,527	4.46
(4) "Hotel" cost component of severe cholera cases hospitalization	1,062,965	0.68	1,062,965	0.83	1,062,965	1.16
(5) Cost cholera-related tests all cholera cases	2,580,675	1.65	2,580,675	2.01	2,580,675	2.81
(6) Health care cost borne by households	434,551	0.28	434,551	0.34	434,551	0.47
***Total Direct Costs***	***8,559,726***	***5.49***	***8,559,726***	***6.68***	***8,559,726***	***9.32***
***(B) Indirect costs***						
(7) Cost of disablement among severe cases	111,551	0.07	111,551	0.09	111,551	0.12
(8) Cost of disablement among mild/moderate cases	919,340	0.59	919,340	0.72	919,340	1.00
(9) Cost of premature deaths	143,984,575	92.30	116,128,266	90.63	79,854,920	86.93
(10) Productivity loss for care-givers	2,418,069	1.55	2,418,069	1.89	2,418,069	2.63
***Total Indirect Costs***	***147,433,535***	***94.51***	***119,577,226***	***93.32***	***83,303,880***	***90.68***
**Grand total cost**	**155,993,261**	**100**	**128,136,952**	**100**	**91,863,606**	**100**

Table 4 depicts three scenario estimates of the direct and indirect costs of cholera in the WHO African Region in 2007. This study estimated the real total economic loss attributable to cholera to have been US$ 43,313,121 assuming a minimum regional life expectancy of 40 years; US$ 59,913,137 assuming a regional average life expectancy of 53 years; and US$ 72,661,207 assuming a maximum regional life expectancy of 73 years. The last estimate was made up of US$4.8 million (6.58%) of direct cost and US$67.9 million (93.42%) of indirect cost. The use of three life expectancies affects only the magnitudes of the productive time lost due to premature death. It can be seen in Tables 2, 3 and 4 that the cost of premature mortality varies directly with life expectancy.

**Table 4:** Direct and indirect real cost of cholera (US$) in 2007 (at 2002 prices)

*Summary of direct costs of cholera*	*Scenario 1: At maximum life expectancy of 73 years*		*Scenario 2: At average life expectancy of 53 years*		*Scenario 3: At minimum life expectancy of 40 years*	
	
	*US$*	% of grand TC	*US$*	% of grand TC	*US$*	% of grand TC
***(A). Direct costs***						
(1) Annual cost of medicines used in treating children and adults suffering mild/moderate cholera state at health centre	140,729	0.19	140,729	0.23	140,729	0.32
(2) Annual cost of medicines used in treating children and adults suffering severe cholera state at hospitals	54,508	0.07	54,508	0.09	54,508	0.13
(3) "Hotel" cost component of mild/moderate cholera cases consultations at health centres	2,080,794	2.84	2,080,794	3.47	2,080,794	4.80
(4) "Hotel" cost component of severe cholera cases hospitalization	510,332	0.70	510,332	0.85	510,332	1.18
(5) Cost cholera-related tests all cholera cases	1,722,805	2.35	1,722,805	2.88	1,722,805	3.98
(6) Health care cost borne by households	271,798	0.37	271,798	0.45	271,798	0.63
***Total Direct Costs***	***4,780,966***	***6.58***	***4,780,966***	***7.98***	***4,780,966***	***11.04***
***(B) Indirect costs***						
(7) Cost of disablement among severe cases	64,184	0.27	64,184	0.11	64,184	0.15
(8) Cost of disablement among mild/moderate cases	532,118	2.27	532,118	0.89	532,118	1.23
(9) Cost of premature deaths	65,892,630	89.90	53,144,560	88.70	36,544,544	84.37
(10) Productivity loss for care-givers	1,391,308	1.03	1,391,308	2.32	1,391,308	3.21
***Total Indirect Costs***	***67,880,240***	***93.42***	***55,132,170***	***92.02***	***38,532,154***	***88.96***
**Grand total cost**	**72,661,207**	**100**	**59,913,137**	**100**	**43,313,121**	**100**

### Average costs

Tables 5, 6 and 7 presents an average real cost per mild/moderate case of cholera, and average cost per severe case of cholera, and average cost per cholera death (US$) in years 2005, 2006 and 2007 respectively. The averages in the three tables were obtained by dividing the itemized total costs in Tables 2, 3 and 4 by the respective year's number of mild/moderate cases, severe cases, and cholera-related deaths.

**Table 5:** Average cost per mild/moderate and severe case of cholera and cholera death (US$) in 2005 (at 2002 prices)

	Scenario 1: Maximum life expectancy of 73 years	Scenario 2: Average life expectancy of 53 years	Scenario 3: Minimum life expectancy of 40 years
	
*Types of costs per person*	*Cost per case (US$)*	*Cost per case (US$)*	*Cost per case (US$)*
(1) Cost of medicines per mild/moderate cholera case	1.6	1.6	1.6
(2) Cost of medicines per severe cholera case	7.0	7.0	7.0
(3) Cost of health centre consultation per mild/moderate cholera case	24.4	24.4	24.4
(4) Cost per hospital admission of per severe cholera case	63.6	63.6	63.6
(5) Cost per cholera test	12.9	12.9	12.9
**(6). Health care cost borne by cholera patient**	2.2	2.2	2.2
(7). Cost of productive time lost per severe cholera case	6.2	6.2	6.2
(8). Cost of productive time lost per mild/moderate cholera case	4.7	4.7	4.7
(9). Cost of productive time lost per premature death	25422.4	20504.0	14099.4
(10). Cost of productive time lost in travel per care giver	11.1	11.1	11.1

**Table 6:** Average cost per mild/moderate and severe case of cholera and cholera death (US$) in 2006 (at 2002 prices)

	Scenario 1: Maximum life expectancy of 73 years	Scenario 2: Average life expectancy of 53 years	Scenario 3: Minimum life expectancy of 40 years
	
*Types of costs per person*	*Cost per case (US$)*	*Cost per case (US$)*	*Cost per case (US$)*
(1) Cost of medicines per mild/moderate cholera case	1.5	1.5	1.5
(2) Cost of medicines per severe cholera case	6.5	6.5	6.5
(3) Cost of health centre consultation per mild/moderate cholera case	22.6	22.6	22.6
(4) Cost of hospital admission per severe cholera case	63.7	63.7	63.7
(5) Cost per cholera test	12.7	12.7	12.7
**(6). Health care cost borne by cholera patient**	2.1	2.1	2.1
(7). Cost of productive time lost per severe cholera case	6.7	6.7	6.7
(8). Cost of productive time lost per mild/moderate cholera case	5.1	5.1	5.1
(9). Cost of productive time lost per premature death	27264.6	21989.8	15121.2
(10). Cost of productive time lost in travel per care giver	11.9	11.9	11.9

**Table 7:** Average cost per mild/moderate and severe case of cholera and cholera death (US$) in 2007 (at 2002 prices)

	Scenario 1: Maximum life expectancy of 73 years	Scenario 2: Average life expectancy of 53 years	Scenario 3: Minimum life expectancy of 40 years
	
*Types of costs per person*	*Cost per case (US$)*	*Cost per case (US$)*	*Cost per case (US$)*
(1) Cost of medicines per mild/moderate cholera case	1.4	1.4	1.4
(2) Cost of medicines per severe cholera case	6.0	6.0	6.0
(3) Cost of health centre consultation per mild/moderate cholera case	20.9	20.9	20.9
(4) Cost of hospital admission per severe cholera case	56.2	56.2	56.2
(5) Cost per cholera test	15.5	15.5	15.5
(6). Health care cost borne by cholera patient	2.5	2.5	2.5
(7). Cost of productive time lost per severe cholera case	7.1	7.1	7.1
(8). Cost of productive time lost per mild/moderate cholera case	5.4	5.4	5.4
(9). Cost of productive time lost per premature death	28811.8	23237.7	15979.2
(10). Cost of productive time lost in travel per care giver	12.6	12.6	12.6

Cost of medicines for mild/moderate cholera case was US$1.4–$1.6; cost of medicines for severe cholera case was US$6.0–$7.0; cost of health centre consultation per mild/moderate cholera case was US$20.9–$24.4; cost of hospital admission per severe cholera case was $56.2–$63.7; cost per cholera test was US$12.7–$15.5; health care cost borne by cholera patient was US$2.1–$2.5; cost of productive time lost per severe cholera case was US$6.2–$7.1; cost of productive time lost per mild/moderate cholera case was US$4.7–US$5.4; and cost of productive time lost during travel per care giver was US$11.1–$12.6. Cost of productive time lost per premature death was US$14,099.4–US$25,422.4 in 2005; US$15,121.2–US$27,264.6 in 2006; and US$15,979.2–US$28,811.8 in 2007.

Tables 8, 9 and 10 provides the average real cost per person with cholera (US$) in 2005, 2006 and 2007 respectively. These averages were obtained by dividing the itemized total costs in Tables 2, 3 and 4 by 125018, 203564 and 110,837 cholera cases in year 2005, 2006 and 2007 respectively. During the three years the average cost of medicines per person with cholera ranged between US$1.8–US$2.1; US$23.4–US$27.1 for provider consultation; US$12.7–US$15.5 for cholera test; US$2.1–US$2.5 for health care cost borne by family; and US$15.8–US$23.7 for productive time lost by patients and family care givers. The average cost of productive time lost due to premature mortality per person with cholera was US$453.5–US$707.3 assuming a maximum regional life expectancy of 73 years; US$365.7–US$570.5 assuming a regional average expectancy of 53 years; and US$251.5–US$392.3 assuming a regional minimum life expectancy of 40 years.

**Table 8:** Average cost per person with cholera (US$) in 2005 (at 2002 prices)

	Scenario 1: Maximum life expectancy of 73 years	Scenario 2: Average life expectancy of 53 years	Scenario 3: Minimum life expectancy of 40 years
	
*Types of costs per person*	*Average cost per person with cholera US$)*	*Average cost per person with cholera (US$)*	*Average cost per person with cholera (US$)*
(1) Cost of medicines	2.1	2.1	2.1
(2) Cost of consultation/hospitalization	27.1	27.1	27.1
(3) Cost of cholera test	12.9	12.9	12.9
(4). Health care cost borne by cholera patient	2.2	2.2	2.2
(5). Cost of productive time lost	15.8	15.8	15.8
(6). Cost of productive time lost due to premature death	453.5	365.7	251.5
(7). Total cost per person with cholera	513.6	425.9	311.6

**Table 9:** Average cost per person with cholera (US$) in 2006 (at 2002 prices)

	Scenario 1: Maximum life expectancy of 73 years	Scenario 2: Average life expectancy of 53 years	Scenario 3: Minimum life expectancy of 40 years
	
*Types of costs per person*	*Average cost per person with cholera US$)*	*Average cost per person with cholera (US$)*	*Average cost per person with cholera (US$)*
(1) Cost of medicines	1.9	1.9	1.9
(2) Cost of consultation	25.3	25.3	25.3
(3) Cost of cholera test	12.7	12.7	12.7
(4). Health care cost borne by cholera patient	2.1	2.1	2.1
(5). Cost of productive time lost	16.9	16.9	16.9
(6). Cost of productive time lost due to premature death	707.3	570.5	392.3
(7). Total cost per person with cholera	766.3	629.5	451.3

**Table 10:** Average cost per person with cholera (US$) in 2007 (at 2002 prices)

	Scenario 1: Maximum life expectancy of 73 years	Scenario 2: Average life expectancy of 53 years	Scenario 3: Minimum life expectancy of 40 years
	
*Types of costs per person*	*Average cost per person with cholera US$)*	*Average cost per person with cholera (US$)*	*Average cost per person with cholera (US$)*
(1) Cost of medicines	1.8	1.8	1.8
(2) Cost of consultation	23.4	23.4	23.4
(3) Cost of cholera test	15.5	15.5	15.5
(4). Health care cost borne by cholera patient	2.5	2.5	2.5
(5). Cost of productive time lost	23.7	23.7	23.7
(6). Cost of productive time lost due to premature death	594.5	479.5	329.7
(7). Total cost per person with cholera	655.6	540.6	390.8

In 2005 the average grand total cost per person with cholera was US$312, US$426 and US$514 assuming a life expectancy of 40, 53 and 73 years respectively. The average grand total cost per person with cholera increased in 2006 to US$451, US$629 and US$766 at 40, 53 and 73 years respectively. In 2007, due to a decrease in notified cholera cases, the average grand total cost decreased to US$391, US$541 and US$656 at 40, 53 and 73 years respectively.

## Discussion

### Key findings

The 125018 cases of cholera notified to WHO by countries of the African Region in 2005 resulted in a total economic loss of US$39 million, US$ 53.2 million and US$64.2 million assuming a regional life expectancies of 41, 53 and 73 years, respectively. The 203,564 cases of cholera notified in 2006 led to a total economic loss US$91.9 million, US$128.1 million and US$156 million assuming life expectancies of 40, 53 and 73 years respectively. The 110,837 cases of cholera notified in 2007 resulted in an economic loss of US$43.3 million, US$60 million and US$77.7 million assuming life expectancies of 40, 53 and 73 years respectively. The main driver of costs of cholera in this study is the premature deaths of young children.

The accuracy these estimates hinges on the plausibility of the assumptions contained in the Appendix [see Additional file 1]; and their interpretation should be tempered with the limitations highlighted below. The reader should keep in mind that the purpose of total cost of illness studies such as the one reported in this paper is not to guide policy decisions but instead to raise awareness among policy-makers and the public about the negative economic impact of cholera. In addition, it is worth noting that many other diarrhoeal, respiratory, and vector borne diseases exacts a greater toll of child mortality than that of cholera.

### Limitations of the study

#### a) Use of the standard treatment guidelines

We have assumed that WHO treatment guidelines for cholera reflect actual practice. In reality this might not be the case. Unfortunately, we did not have information on what fraction of cases actually received the standard treatment and what proportion did not. In addition, we were not in a position to speculate the implications of non-adherence on the total cost of cholera illness.

#### b) Assumption that those who suffer cholera mortality would have future earning

In this study we assumed that all those who die from cholera would have future earnings. This assumption can be contested especially in African countries where the formal sectors are small and hence the proportion of people in formal employment is small. In situations where unemployment rate is high, the marginal productivity of labor might be less than the average. Should this be the case, the use of gross national income per capita might over-estimate the economic burden of cholera.

#### c) Assumption that all cholera cases receive a diagnostic test

We have assumed that all cholera cases receive a diagnostic test. In reality, this might not be the case. We used an average cost obtained from a number of countries in the African Region. By so doing we may have overestimated the cost of diagnosis.

#### d) Omission of intangible costs

The majority of the data used in this study were obtained from secondary sources due to research resource constraints. Therefore, it was not possible to conduct a household survey that would have made it possible to estimate the intangible costs using contingent willingness-to-pay approach [21].

#### e) Use of per capita GNI to value-productive time lost

This study attempted to estimate the loss in the gross national income (GNI) and not the total economic cost of cholera morbidity and premature mortality. The social value of the contribution that women make to African societies is greater than that captured in GNI calculations. This is because the International Labour Organization's (ILO) definition of labour force includes the employed (including the armed forces), the unemployed, and the first-time job-seekers, but excludes full-time homemakers and other unpaid caregivers and workers in the informal sector. The majority of the women in Africa are either full-time homemakers and/or informal sector workers, and, thus, their invaluable contribution to society is excluded from GNI calculations [22,23].

#### f) Omission of economic effects of cholera outbreaks on tourism and commodity export

We mentioned in the **Methods **section that when there are cholera outbreaks in African countries, developed countries usually discourage their citizens from traveling to the affected countries, which reduces the number of tourists to those countries. Consequently, that leads to losses of revenues for the tourism industry, unemployment of people whose livelihood is dependent on tourism, and reduction in tax revenues for the governments. In addition, usually, there is an international embargo on export of commodities from countries experiencing cholera outbreaks. When the latter happens, it may adversely affect the foreign exchange flow into the affected countries, which is likely to have many other externality costs.

## Conclusion

In spite of data limitations, the estimates reported here show that cholera imposes substantial economic burden on countries of the African Region. That heavy burden underscores the urgent need for increased investments in the prevention and control of cholera.

In addition, there is an urgent need for further research to determine:

• national-level economic burden of cholera, broken down by different productive and social sectors and occupations of patients and relatives; and

• national-level costs, effectiveness and monetary benefits of alternative ways of scaling up population coverage of appropriate potable water and clean sanitation facilities in rural, peri-urban and urban areas.

## Competing interests

The authors declare that they have no competing interests.

## Authors' contributions

JMK, LGS, AY, ES, LKM and DGK participated equally in the design, analysis and writing of the manuscript. All the authors read and approved the final manuscript.

## Pre-publication history

The pre-publication history for this paper can be accessed here:

http://www.biomedcentral.com/1472-698X/9/8/prepub

## Supplementary Material

Additional file 1 **Appendix: Assumptions underpinning the estimations of economic burden of cholera**. The file contains the assumptions used in the economic burden estimations. Click here for file
