# Supplementary material for: Economic burden of cholera in the WHO African region
Source: BMC Int Health Hum Rights. 2009 Apr 30;9:8. doi: 10.1186/1472-698X-9-8 (PMC2691726; doi:10.1186/1472-698X-9-8)
Supplement: Additional file 1 — Appendix: Assumptions underpinning the estimations of economic burden of cholera. The file contains the assumptions used in the economic burden estimations. [file 1472-698X-9-8-S1.doc]

**Appendix: Assumptions underpinning the estimations of economic burden of cholera**

| Variable | Value | Source |
| --- | --- | --- |
| Probability of death at different age brackets | Age 0-4 = 0.851578947;  Age 5-14 = 0.074736842;  Age 15-44=0.029473684;  Age 45-59=0.009473684;  Age 60+= 0.034736842. | Murray and Lopez [20] |
| Average age of onset (in years) of cholera | Age 0-4 =2.4;  Age 5-14 =10;  Age 15-44=29.45;  Age 45-59=52.2;  Age 60+=69.46. | Murray and Lopez [20] |
| Average life expectancy at birth | Minimum=40 years in Sierra Leone; Regional Average=53 years; maximum=73 years in Mauritius | WHO [16] |
| Average gross national income per capita for African Region | US$1311 in 2005; US$1518 in 2006; US$1730 in 2007 | World Bank [15] |
| Average number of days lived with mild/moderate cholera | 4 | WHO [11] |
| Average duration (in years) lived in severe cholera state | 0.02 | Lopez and Murray [20] |
| Total number of cholera cases notified to WHO | 125018 cases in 2005; 203564 cases in 2006; 110837 cases in 2007. | WHO [8] |
| Total number of cholera deaths | 2230 deaths in 2005; 5281 deaths in 2006; 2287 deaths in 2007. | WHO [8] |
| Number of persons accompanying a patient to a health facility | 1 | Kirigia and Sambo [19] |
| Number of health facility visits per year per person | 3 | WHO [11] |
| Average length of stay (days) at an hospital | 3 | WHO [11] |
| Distribution of cholera cases across mild/moderate, severe and dead states | Mild/moderate=0.900162569; severe=0.082; deaths=0.018 | Murray and Lopez [20] |
| Distribution of cholera cases across age groups | Age 0-4=0.6803; Age 5-14=0.1930; Age 15-44=0.0965; Age 45-59=0.0195; Age 60+=0.0107 | Murray and Lopez [20] |
| Unit price of medicine for a child and adult at a health centre | US$0.25 | WHO/AFRO [12] |
| Cost of medicines at hospital for children & adults | US$9 per child; US$9.91 per adult | WHO [9] |
| Cost of a consultation among mild/ moderate patients at health centre | US$10.92 | WHO [9] |
| Unit cost per bed day at levels 1, 2 & 3 hospitals | US$23.42 | WHO [9] |
| Cost per cholera test | US$17.38 in 2005; US$18.41 in 2006; US$24 in 2007 | A quick survey by WHO Country Office health economists in 10 countries. |
| Average cost borne by an household (consultation, medicines, tests, transport, other) | US$2.94 in 2005; US$3.1 in2005; US$3.84 in 2007 | WHO [13] and IMF [14] |
| Discount rate (%) | 3 | WHO [17] |
| Regional average consumer price index | Year 2002=174; year 2005=235; year 2006=253; year 2007=273 | IMF [14] |
